# Reliability of durometry to assess firmness of calcinosis lesions in Juvenile and adult dermatomyositis

**DOI:** 10.1371/journal.pone.0343708

**Published:** 2026-03-23

**Authors:** Meghan Corrigan Nelson, Lisa G. Rider, Hanna Kim, Scott Gillespie, Vy Do, Julie Fuller, Kelly Rouster-Stevens, Adam Schiffenbauer

**Affiliations:** 1 Division of Pediatric Rheumatology, Department of Pediatrics, Emory University School of Medicine, Atlanta, Georgia, United States of America,; 2 Children’s Healthcare of Atlanta, Atlanta, Georgia, United States of America,; 3 National Human Genome Research Institute, National Institutes of Health, Bethesda, Maryland, United States of America; 4 National Institute of Arthritis and Musculoskeletal and Skin Diseases, National Institutes of Health, Bethesda, Maryland, United States of America; 5 Environmental Autoimmunity Group, Clinical Research Branch, National Institute of Environmental Health Sciences, National Institutes of Health, Bethesda, Maryland, United States of America; 6 Juvenile Myositis Pathogenesis and Therapeutics Unit, National Institute of Arthritis and Musculoskeletal and Skin Diseases (NIAMS), NIH, Bethesda, Maryland, United States of America; 7 Emory University, Atlanta, GeorgiaUnited States of America; 8 Texas Scottish Rite Hospital for Children, Dallas, Texas, United States of America; 9 Department of Pediatrics, University of Texas Southwestern Medical Center, Dallas, Texas, United States of America; Faculty of Medical Sciences of Minas Gerais, BRAZIL

## Abstract

**Background/Objective:**

Dermatomyositis (DM) and juvenile dermatomyositis (JDM) are inflammatory myopathies affecting multiple organs, including muscle and skin. Calcinosis is a complication of DM/JDM that causes significant morbidity; however, few tools exist to assess calcinosis in DM/JDM patients This study aimed to evaluate the reliability of durometry measurements to assess the firmness of calcinosis lesions in DM and JDM patients.

**Methods:**

Calcinosis firmness was measured using a handheld digital durometer. Six investigators across 3 institutions examined DM/JDM calcinosis lesions by durometry, as well as control readings in healthy unaffected skin/subcutaneous tissue in similar anatomic areas, recording three readings per site. Intra-rater and inter-rater intraclass correlations were evaluated.

**Results:**

We enrolled 57 patients and gathered 709 measurements (443 calcinosis lesions; 266 control lesions) over eleven anatomic regions. Intra-rater reliability was high across sites, while inter-rater reliability varied, being moderate to good in most areas, but poor in the thigh and anterior calf. Durometry readings were higher in calcinosis lesions than control sites overall. Measurements moderately correlated with qualitative physician assessments. Due to our study’s cross-sectional nature, we could not assess calcinosis over time.

**Conclusion:**

Durometry is a novel, reliable, quantitative measure in assessing and characterizing targeted calcinosis lesions of DM/JDM patients.

## Introduction

Dermatomyositis (DM) and juvenile dermatomyositis (JDM) are idiopathic inflammatory myopathies characterized by proximal skeletal muscle inflammation and skin manifestations [1]. Calcinosis, a complication of DM/JDM, is associated with significant morbidity and worse functional outcomes [1–5]. Calcinosis can present as superficial lesions, deep nodules, or deposits along myofascial planes, with varying firmness [5].

Durometers are handheld devices used to measure firmness by applying a load on various surfaces [6]. In scleroderma, durometry has been used as a measure of skin fibrosis [6–8]. Durometry presents a viable tool for patient encounters, as measurements can be done rapidly and typically involve no discomfort [6]. In addition, studies have found that skin firmness is relatively constant in healthy control subjects between 15 and 65 years of age, allowing for relatively consistent normal values [8].

While previous studies have evaluated the role of durometry in measuring skin firmness in scleroderma, studies assessing durometry in measuring calcinosis are limited [9,10]. While recent studies have shown changes in durometry readings of calcinosis lesions following treatment of JDM patients, the reliability of these measurements has not been addressed and is an important step in validating the use of durometry [9,10]. Durometry may serve as a useful quantifiable tool for the assessment of calcinosis firmness in patients with DM/JDM, which would allow calcinosis firmness to be evaluated as a potential outcome measure in therapeutic studies of calcinosis. Full validation of durometry as an outcome assessment tool requires demonstration of reliability and sensitivity by multiple investigators. The goal of this study was to determine the reliability of repeated calcinosis durometer measurements in DM/JDM patients, both within providers (intra-rater) and between providers (inter-rater), as well as to further characterize calcinosis lesions within our patient cohort.

## Materials and methods

### Subjects

Patients with DM/JDM meeting probable or definite Bohan and Peter classification criteria [11] and probable or definite ACR/EULAR classification criteria [12] with calcinosis by imaging and/or physician examination were enrolled across three centers. Based on preliminary data, a minimum of 50 patients was determined to be sufficient sample size to capture clinical differences between patients. Sites of enrollment included the National Institutes of Health (NIH, Bethesda, MD), Children’s Healthcare of Atlanta (CHOA, Atlanta, GA) and Texas Scottish Rite Hospital for Children (TSRHC, Dallas, TX) from 01/12/2015–01/08/2021. Data used in this study was accessed for primary research analyses 01/04/2022 through approximately 09/25/2023. Demographic data, current medications, and assessments of disease activity and damage were obtained at enrollment. Serum from 45 patients was tested for 5 myositis specific autoantibodies (MSAs) and 3 myositis associated autoantibodies (MAAs) by standard immunoprecipitation [13] and immunoprecipitation-immunoblotting methods [14]. Ethnicity was self-reported by questionnaire.

### Ethics

Patients were enrolled in a myositis natural history study (protocol 94E0165, NCT00017914) approved by the NIH institutional review board; each enrolling site also obtained local IRB approval with written consent (CHOA,TSRHC) and conducted according to the principles expressed in the Declaration of Helsinki. All patients and parents signed informed consent. The data were fully anonymized before access by the research team.

### Durometer calcinosis hardness scores

Durometer measurements were made using a handheld digital durometer (Rex Gauge 1600 Type DD-4–00 Durometer, Buffalo Grove, IL) (S1 Fig), which uses a continuous scale measured in standardized durometer units (DU). Standard techniques were developed and durometry was performed similarly across all three centers. Three consecutive durometer measurements were taken at each calcinosis lesion and anatomically corresponding control sites. Control regions were obtained on non-affected mirrored contralateral sides, where applicable. Measurements were made with the skin in a horizontal plane. Measurements were obtained by placing the durometer laterally and rolling it onto the lesion, in order to be measured consistent with prior durometry studies [8]. The measurements were recorded in a standardized form along with their anatomic location, which was based on 60 anatomic location codes. Location codes were to be reviewed for lesions per area and location similarity and combined into larger areas before analysis (S1 Form). Notably no patients reported pain that limited or restricted the examination. Only gravitational force was applied during measurements, with no additional pressure, thereby eliminating variability due to examiner-applied force.

Intra-rater reliability was assessed by having a single designated (primary) rater, a rheumatology-trained physician, perform durometer measurements on each calcinosis lesion at a single study visit, with repeat measurements performed either at the time of initial examination or within 24 hours of the initial assessment. Dr. Rider evaluated patients < 18 years at the NIH Clinical Center, while Dr. Schiffenbauer assessed those ≥18, unless a preexisting investigator-patient relationship designated another primary rater. Primary raters at CHOA and TSRHC were identified as the patients’ treating rheumatologist. Inter-rater reliability was assessed only at the NIH. Inter-rater reliability was assessed by comparing measurements from a primary rater to measurements from a secondary rater(s). Lesions were demarcated with a skin marking pen at the targeted sites. Raters performed their assessments blinded to and within 5 days of the other raters’ assessments. Intra- and inter-rater reliability were calculated when five or more assessments were available per anatomic location. When available, calcinosis lesions were qualitatively characterized as hard, wooden, fluctuant, or liquified (S1 Form).

### Statistical analyses

All analyses were performed in SAS v.9.4 (SAS Institute, Cary NC) and CRAN R v.4.2 (R Core Team 2022), and statistical significance was set at the 0.05 threshold. Demographic and clinical features for DM/JDM patients were summarized using medians [interquartile ranges, IQR] or means ± standard deviations (SD) (range) for continuous variables and frequencies (percentages, %) for categorical variables.

Primary outcomes (lesion measurements) were treated as continuous variables and analyzed using ICCs and general linear regression methods with maximum likelihood estimation. The primary exposure, calcinosis versus control lesion site (categorical, 2-level), was examined, with anatomic location (categorical, 11-level) considered as an effect modifier. Therefore, analyses were stratified by anatomic location, and comparisons between calcinosis and control lesion sites were made when applicable. Missing data were assumed to be missing at random (MAR). No imputation was performed; instead, maximum likelihood estimation was used in all regression models to prevent listwise deletion of records with missing data. The ICC methods applied in the analysis to accommodate missing data are detailed below.

Intra- and inter-rater reliability were calculated using intraclass correlations (ICCs). Using guidance from Koo & Li (2016), intra-rater ICCs were derived using single-rater, two-way mixed effects models assessing for absolute agreement, and treating raters as fixed effects and the measurements as random effects [15]. For inter-rater ICCs, absolute agreement, two-way random effects models were utilized, treating both raters and measurements as random effects. Fixed effects were specified for raters in the intra-rater ICC models, as it is not reasonable to generalize one rater’s scores against themself to a population of raters. Conversely, random effects were specified for raters in the inter-rater ICC models, as the goal was to generalize results to other raters in a real-world setting. All ICCs were accompanied by 95% Wald confidence intervals (CIs). Repeated measurements at the same location for the same rater were averaged for inter-rater analysis. Inter-rater calculations were then performed, comparing averaged durometry assessments from the primary rater to the averaged durometry assessments from the secondary rater. In some situations, measurements from up to 2 secondary raters were available for a single primary rater measurement. These data imbalances were circumvented by use of the *irrNA* package (v.0.2.2) in CRAN R, which calculates ICCs based on continuously scaled data and derives reliability estimates in the presence of imbalanced data without imputation or listwise deletion. Inter-rater ICC values were also run using initial observations, instead of averages, for sensitivity analysis. Given the novel use of durometry in DM/JDM without prior published data regarding its use in this patient population, intra-rater and inter-rater ICCs were interpreted based on prior published generalized cut-offs: excellent (greater than 0.9), good (0.75 to 0.9), moderate (0.5 to 0.75), and poor (less than 0.5) [16]. Finally, a general linear mixed model with raters treated as fixed effects was used to determine if paired calcinosis and control measurements significantly differed by location. Results are reported as least-squares means (LS-means) and LS-mean differences with 95% CIs, p-values, and ranges of the data. Our methods were reviewed by a second independent statistical team who provided feedback on our analyses.

## Results

Fifty-seven patients were enrolled in the study, 30 (55%) had JDM, and 27 (45%) had DM. There was a predominance of Caucasian (58%) and female (63%) individuals with a median age of 18 years (inter-quartile range 14–30 years) (Table 1). A total of 45 patients were assessed at the NIH, 8 patients were assessed at CHOA, and 4 patients were assessed at the TSRHC. There were 3 raters (NIH), 1 rater (CHOA), and 1 rater (TSRHC) available for assessments, respectively. Overall, there were 129 patient-rater interactions, and 11 unique anatomic location codes, for a total of 398 independent lesions with 709 durometry assessments of both calcinosis and control lesions. There were 33 patients with 176 durometry measurements who had multiple assessors that could be used for inter-rater reliability analysis; of those, 112 (64%) had 3 assessors and 64 (36%) had 2 assessors.

**Table 1:** Demographic and Clinical Features of 57 Adult and Juvenile Dermatomyositis Patients in the Study.

Characteristic	Mean ± SD (Range), or N (%)
**Age, years**	18 [14, 30]
**Race**	
White	33 (57.8%)
Black	16 (28.1%)
Asian	3 (5.3%)
Native American	1 (1.8%)
Multiple Races	4 (7.0%)
**Ethnicity**	
Not Hispanic/Latino	46 (80.7%)
Hispanic/ Latino	11 (19.3%)
**Sex** (N = 57)	
Female	36 (63.2%)
**Current Medications**	
Prednisone	34 (59.6%)
Methotrexate	26 (45.6%)
IVIG	24 (42.1%)
Hydroxychloroquine	18 (31.6%)
Mycophenolate mofetil	14 (24.6%)
Intravenous Methylprednisolone	12 (21%)
Leflunomide, Tacrolimus or Cyclosporine	6 (10.5%)
TNF inhibitors	3 (5.3%)
Rituximab	3 (5.3%)
**Average number of DMARDs**	1.2 ± 0.8 (0-3)
**Average number of Biologics**	0.5 ± 0.6 (0-2)
**Disease Course**	
Monocyclic	2 (3.5%)
Chronic Continuous	38 (66.7%)
Chronic Polycyclic	16 (28.1%)
Undefined	1 (1.8%)
**Autoantibody Status**	
Positive Myositis Specific Autoantibody^1^	35 (61.4%)
Positive Myositis Associated Autoantibody^2^	9 (15.8%)
**Disease Activity and Damage Indices**	
Physician Global Activity, 0–10 cm VAS	3.3 ± 2.5 (0-9.3)
Adjusted Calcinosis Severity, 0–10 cm VAS	3.6 ± 2.5 (0-9.6)
CMAS Score (N = 54)	42.7 ± 9.9 (13-52)
MDI Total Severity Score (0–110) (N = 41)	13.8 ± 9.9 (2-39)
MDI Cutaneous Damage, 0–10 cm VAS (N = 41)	4.7 ± 2.6 (0.9-9.7)

^1^Myositis Specific Autoantibodies (MSA) included anti-MJ/NXP2 (23 patients), -Jo1 (1), -MDA5 (5), -Mi2 (1), or -P155 (TIF1) autoantibodies (5); 8 patients were MSA negative and 14 patients did not have MSAs assessed

^2^Myositis Associated Autoantibodies (MAA) included Anti-PM/ScL (3), -Ro (4), or -U1RNP autoantibodies (2); 36 patients were MAA negative and 12 patients did not have MAAs assessed

DMARDs comprise: methotrexate, hydroxychloroquine, azathioprine, leflunomide, mycophenolate mofetil, cyclosporin, tacrolimus; Biologics comprise: TNF (Tumor Necrosis Factor) inhibitors adalimumab or infliximab, IVIG (Intravenous Immunoglobulin), rituximab

Abbreviations: DMARDs, Disease Modifying Anti-Rheumatic Drugs, MDI, Myositis Damage Index; CMAS, Childhood Myositis Assessment Scale; VAS, Visual Analog Scale

The median Physician Global Activity was 3.3 ± 2.5 and the median Childhood Myositis Assessment Score was 42.7 ± 9.9 (Table 1). Patients also had damage, indicated by the average Myositis Damage Index (MDI) severity of 13.8 ± 9.9 (Table 1). Approximately 67% of patients had a chronic continuous disease course, and only 3% had a monocyclic course (Table 1).

A total of 709 durometric measurements were obtained, consisting of 443 calcinosis and 266 control lesion measurements, and included 244 paired calcinosis and control assessments from 60 specific locations. The range of measurements for calcinosis lesions was 2.2 to 87.1 durometry units (DU) and the range for control sites was 1.6 to 73 DU. Anatomic sites that were similar in location and with similar underlying bony architecture were combined, such that the 60 anatomic location codes were condensed to 11 anatomic sites. The 11 anatomic sites included upper neck/clavicle, back/torso, upper arms bilaterally, forearms bilaterally, elbows, hands/wrists, buttocks, thigh, anterior calf, posterior calf, and feet (S1 Table). All patients were able to complete the examination, and no injuries were reported.

Overall, our study indicated good to excellent intra-rater reliability in durometry measurements (Table 2). For calcinosis sites, intra-rater ICCs ranged from 0.75–0.93, with 4 anatomic sites demonstrating excellent ICCs (thigh, elbows, hands/wrists, and foot) and 7 sites demonstrating good ICCs (back/torso, posterior calf, buttocks, anterior calf, upper arms, upper neck/clavicle, and forearms).

**Table 2:** Intra-rater and inter-rater for calcinosis and control lesions for DM/JDM patients by anatomic location.

Anatomic Location^1,2^	Intra-rater Assessments^3^	Intra-rater ICC(95% CI)	Inter-rater Assessments^4,5^	Inter-rater ICC(95% CI)
**Calcinosis Location**	n = 443		n = 113	
Upper Neck/Clavicle	12	0.82 (0.60, 0.94)	2	--
Back/Torso	53	0.90 (0.85, 0.94)	15	0.48 (0.13, 0.76)
Upper Arms	88	0.85 (0.80, 0.90)	27	0.76 (0.59, 0.87)
Forearms	32	0.75 (0.60, 0.86)	9	0.64 (0.25, 0.89)
Elbows	51	0.92 (0.88, 0.95)	9	0.85 (0.61, 0.96)
Hands/Wrists	26	0.92 (0.86, 0.96)	4	--
Buttocks	14	0.87 (0.73, 0.95)	3	--
Thigh	105	0.93 (0.90, 0.95)	26	0.32 (0.05, 0.58)*
Anterior Calf	38	0.86 (0.77, 0.92)	10	0.30 (−0.16, 0.73)*
Posterior Calf	16	0.90 (0.79, 0.96)	6	0.79 (0.40, 0.96)
Foot	8	0.91 (0.74, 0.98)	2	--
**Control Location**	n = 266		n = 63	
Upper Neck/Clavicle	9	0.85 (0.63, 0.96)	1	--
Back/Torso	29	0.81 (0.68, 0.90)	9	0.65 (0.22, 0.90)
Upper Arms	53	0.91 (0.86, 0.94)	16	0.47 (0.13, 0.75)
Forearms	35	0.95 (0.92, 0.97)	9	0.83 (0.55, 0.95)
Elbows	24	0.97 (0.93, 0.98)	4	--
Hands/Wrists	9	0.85 (0.57, 0.96)	0	--
Buttocks	9	0.77 (0.47, 0.94)	2	--
Thigh	54	0.90 (0.84, 0.93)	12	0.30 (−0.12, 0.70)
Anterior Calf	29	0.91 (0.84, 0.95)	5	0.56 (−0.03, 0.93)
Posterior Calf	10	0.63 (0.29, 0.88)	4	--
Foot	5	0.81 (0.40, 0.98)	1	--

^1^57 patients and 5 raters provided data for 129 patient-provider interactions, on 11 unique anatomic location codes and for a total of 398 independent lesions, totaling 709 durometry measurement assessments of both calcinosis and control lesions.

^2^Intra-rater and inter-rater ICCs calculated when the number of assessments was ≥ 5.

^3^Each assessment was generally comprised of 3 repeated durometry measurements from the same rater for intra-rater ICC calculation. Multiple individuals may have assessed the same lesion. Assessments were performed for a total of 398 independent lesions, of which 244 were calcinosis lesions and 154 were control locations.

^4^Repeated intra-rater durometry measurements were averaged and compared between corresponding primary and secondary raters, when available, for inter-rater ICC evaluation. This included assessments from three raters at the National Institutes of Health (Bethesda, MD), one rater from Children’s Healthcare of Atlanta (Atlanta, GA) and one rater from Texas Scottish Rite Hospital for Children (Dallas, TX).

^5^176 primary assessments had ≥ 1 corresponding secondary assessments for inter-rater ICC calculations (n = 176 inter-rater pairings, comprised of 464 primary and secondary assessments).

* Indicates ICC level below the acceptability threshold.

Abbreviations: DM] (Dermatomyositis); JDM (Juvenile Dermatomyositis); ICC (Intraclass Correlation Coefficient); 95% CI (Confidence Interval).

Since intra-rater results were strong, repeated measurements at the same location from the same rater were averaged prior to inter-rater analysis. Inter-rater reliability could not be calculated for four calcinosis locations and six control locations due to too few observations (< 5 measurements). In the subset of patients with inter-rater data available, inter-rater reliability ranged from good to poor, depending on anatomic location. Inter-rater ICCs spanned from 0.30 to 0.85 (Table 2). For calcinosis lesions, the upper arms, elbows, and posterior calf had good inter-rater ICCs. For control lesions, forearms had a good inter-rater ICC. The sensitivity analysis performed for inter-rater ICC values using the first durometry observation rather than averaged durometry values yielded overall similar results, except the anterior calf where the ICC difference was 0.21 (S3 Table).

Significant differences in durometry measurements were seen between calcinosis and control sites. Paired analyses of measurements for calcinosis versus control sites were significantly different, with greater LS-mean measurements for calcinosis sites relative to control sites at each anatomic location (all p < 0.01, Table 3). Notably, the locations with the highest LS-mean difference between calcinosis and control sites were forearms (LS-mean difference: 31.6), upper arms (28.1), and foot (24.4). Elbows (10), posterior calf (15.8), and upper neck/clavicle (16.9) had the smallest LS-mean difference between calcinosis and control sites (Table 3).

**Table 3:** Paired mixed model least-squares mean (LS-means) differences in durometry measurements between calcinosis and control lesions, by anatomic location.

Anatomic Location^1,2^	N Paired Assess. N = 244	LS-Mean^3^ (95% CI)	P-Value	Range
**Upper Neck/Clavicle**				
Calcinosis	9	28.4 (20.4, 36.4)		2.2 - 56.5
Control		11.5 (3.5, 19.5)		2.7 - 25.6
Difference (Calcinosis – Control)		16.9 (7.1, 26.7)	**0.001**	
**Back/Torso**				
Calcinosis	29	27.5 (23.1, 32.0)		2.2 - 46.7
Control		9.9 (5.5, 14.4)		1.6 - 22.8
Difference (Calcinosis – Control)		17.6 (12.2, 23.1)	**<0.001**	
**Upper Arms Bilaterally**				
Calcinosis	48	40.5 (37.1, 44.0)		16.8 - 67.3
Control		12.4 (9.0, 15.9)		1.8 - 39.2
Difference (Calcinosis – Control)		28.1 (23.9, 32.3)	**<0.001**	
**Forearms Bilaterally**				
Calcinosis	24	50.7 (45.8, 55.6)		26.7 - 87.1
Control		19.1 (14.2, 24.0)		4.7 - 30.7
Difference (Calcinosis – Control)		31.6 (25.6, 37.6)	**<0.001**	
**Elbows**				
Calcinosis	24	37.8 (32.9, 42.7)		9.8 - 65.7
Control		27.8 (22.9, 32.7)		2.4 - 61.5
Difference (Calcinosis – Control)		10.0 (4.0, 15.9)	**0.001**	
**Hands/Wrists**				
Calcinosis	9	44.7 (36.7, 52.7)		29.3 - 61.8
Control		23.6 (15.6, 31.6)		17.8 - 33.6
Difference (Calcinosis – Control)		21.0 (11.2, 30.8)	**<0.001**	
**Buttocks**				
Calcinosis	7	26.3 (17.2, 35.3)		10.2 - 34.2
Control		7.3 (−1.7, 16.4)		3.6 - 11.4
Difference (Calcinosis – Control)		18.9 (7.8, 30.0)	**0.001**	
**Thigh**				
Calcinosis	52	36.9 (33.6, 40.3)		6.6 - 72.5
Control		17.7 (14.4, 21.0)		4.3 - 47.2
Difference (Calcinosis – Control)		19.2 (15.2, 23.3)	**<0.001**	
**Anterior Calf**				
Calcinosis	27	46.2 (41.6, 50.8)		20.2 - 76.3
Control		25.9 (21.2, 30.5)		13.8 - 56.0
Difference (Calcinosis – Control)		20.4 (14.7, 26.0)	**<0.001**	
**Posterior Calf**				
Calcinosis	10	35.6 (28.0, 43.2)		16.8 - 56.8
Control		19.8 (12.2, 27.4)		12.1 - 25.7
Difference (Calcinosis – Control)		15.8 (6.5, 25.1)	**0.001**	
**Foot**				
Calcinosis	5	47.0 (36.3, 57.7)		34.3 - 62.3
Control		22.6 (11.9, 33.4)		17.5 - 37.3
Difference (Calcinosis – Control)		24.4 (11.2, 37.5)	**<0.001**	

^1^57 patients and 5 raters provide data for 129 patient-provider interactions, on 11 unique location codes and for a total of 398 independent lesions totaling 709 durometry measurement assessments of both calcinosis and control lesions.

^2^Intra-rater and inter-rater ICCs calculated when the number of assessments was ≥ 5.

^3^Repeated intra-rater durometry measurements were averaged and these averages were used as raw data inputs into the general linear mixed effects regression models.

Abbreviations: DM (Dermatomyositis); JDM (Juvenile Dermatomyositis); ICC (Intraclass Correlation Coefficient); 95% CI (Confidence Interval)

For primary rater’s qualitative descriptors the most frequently reported characteristic of calcinosis lesions was ‘hard’ followed by ‘wooden’ and the least frequently reported was ‘fluctuant/liquified’(S2 Table). Overall, ‘fluctuant/liquified’ calcinosis lesions were associated with lower durometry measurements [mean 32.1 ± 13.3 DU], compared to ‘hard’ calcinosis lesions which were associated with the highest durometry measurements [mean 42.7 ± 16.7 DU] (Table 4). Intra-rater ICCs were generally good to excellent for all hard, wooden, fluctuant/liquified, and combined lesions (S2 Table).

**Table 4:** Characteristics of calcinosis lesions and corresponding durometry measurements in patients with DM and JDM at initial visit^1^.

Characteristics of Lesions	n (%)	Durometry MeasurementsMean ± SD (Range)
Hardened Only	90 / 169 (53.3%)	42.7 ± 16.7 (14, 87.1)
Wooden Only	12 / 169 (7.1%)	31.9 ± 9.7 (15.5, 43.2)
Fluctuant/Liquified Only	7 / 169 (4.1%)	32.1 ± 13.3 (16.8, 53.2)
All Characteristics	12 / 169 (7.1%)	42.5 ± 16.8 (15.9, 70.4)
Hardened + Wooden	40 / 169 (23.7%)	41.7 ± 15.2 (10, 75.1)
Wooden + Fluctuant/Liquified	8 / 169 (4.7%)	29.6 ± 18.5 (2.2, 53.6)

^1^Durometry reading sample sizes and summaries by calcinosis lesion characteristics in primary rater. ^2^

^2^Patients N = 40, number of lesions n = 166.

Abbreviations: DM (Dermatomyositis); JDM (Juvenile Dermatomyositis); SD (Standard Deviation).

## Discussion

This study demonstrates the reliability and content validity of durometry in evaluating calcinosis, and helps to quantifiably characterize calcinosis in DM/JDM patients. Our study shows viability of durometry as a measure of calcinosis given our high intra-rater reliability and predominantly moderate to good inter-rater reliability, although there was noted poor inter-rater reliability noted at the thigh and anterior calf. Additionally, this study confirms durometry’s content validity with greater firmness of lesions among pooled calcinosis versus control sites across anatomic regions. There is also evidence for the construct validity of durometry, as qualitative descriptions of durometry correlate with noted variations observed in quantitative durometry measurements (Table 4, S2 Table). Moreover, there is evidence for face validity, given the direct measurement of calcinosis by this tool in DM (Table 2), as well as its historical use in scleroderma as an effective tool in measuring skin firmness [5–8].

Overall, our results suggest reliable measures of durometry in DM/JDM patients, and therefore durometry could be a potentially useful tool in clinical practice and future therapeutic trials for calcinosis. Our inter-rater agreement showed a majority of assessments indicating moderate to good reliability with variation between different areas. Both calcinosis and control inter-rater reliability in the thigh were decreased. This suggests that there may be an inherent heterogeneity of durometry readings in this anatomic location. Sensitivity analysis also revealed decreased inter-rater ICC for the anterior calf. This heterogeneity by anatomic site highlights the importance of picking an appropriate site for evaluation. Of note, the elbows offered excellent intra-rater reliability, good inter-rater reliability, and are a relatively common site of calcinosis in DM/JDM patients.

Limitations of this study include small sample sizes for some anatomic regions, limiting statistical comparisons, and that inter-rater comparisons were only performed at one study site. While our study utilized three academic centers across the United States, future studies should increase the number of participants and centers. This study examined inter-rater reliability, capturing variability between users, which was moderate to good in most anatomic locations. Given that providers had limited experience with durometers prior to this study, additional training may further improve inter-rater agreement. Overall, the differences in anatomic location were subtle and should be substantiated with further studies. Caution should be used when selecting potential sites for measurement of calcinosis durometry readings due to aforementioned variability in readings. Another limitation is absence of data from serial evaluation and limited lesion size data. Due to the limited physical area detected by durometry at a given time, it would not be practical to detect calcinosis in a large anatomic area; however, we were able to successfully capture characteristics of calcinosis due to firmness in a more targeted area, where it helped determine calcinosis versus control status. Furthermore, durometry has not been evaluated for predictive validity. Additionally, there is a need to correlate durometry measurements with clinical outcome measures and disease progression.

Overall, our study identifies durometry as a reliable, quantitative tool in assessing calcinosis in DM/JDM patients, of particular importance given the current lack of quantifiable measures to assess calcinosis firmness. Broad implementation of durometry across institutions could enable more robust comparisons among clinical trial cohorts and future observational studies. Durometry offers a tool to objectively assess calcinosis during patient encounters, with potential utility in providing an objective and reliable measurement of calcinosis for clinical trials. We demonstrated content validity in DM/JDM patients given greater firmness of lesions among calcinosis versus control sites by anatomic regions, as well as acceptable reliability. While durometry is a promising method for quantitative assessment of cutaneous calcinosis in DM/JDM, additional studies with measurement over time and relationship to clinical outcomes are needed to further validate durometry and substantiate its role in the assessment of calcinosis in DM/JDM patients.

## Supporting information

S1 Fig Image of Handheld Digital Durometer. Similar make and model of the handheld digital durometer (Rex Gauge 1600 Type DD-4-00) utilized for quantitative assessment of calcinosis firmness.
(DOCX)

S1 Form Calcinosis Type – Sentinel Lesion Form. Categorization of calcinosis lesions by type and anatomic location, were recorded in this Sentinel Lesion Form.
(DOCX)

S1 Table Collapsed anatomic sites derived from Sentinel Lesion Form. Detailed anatomic site responses from the Sentinel Lesion Form were reviewed and combined into harmonized categories to facilitate downstream analyses. This table summarizes each collapsed site grouping and its component locations.
(DOCX)

S2 Table Intra-rater ICCs and 95% CI for calcinosis assessments by density characteristics for DM/JDM patients. This table presents intra-rater intraclass correlation coefficients (ICCs) and corresponding 95% confidence intervals for calcinosis assessments stratified by density characteristics in patients with dermatomyositis and juvenile dermatomyositis (DM/JDM).
(DOCX)

S3 Table Sensitivity analysis for inter-rater reliability of calcinosis and control assessments by Anatomic Location for DM/JDM patients. This table summarizes sensitivity analyses of inter-rater agreement for calcinosis and control assessments across specific anatomic locations in DM/JDM participants, providing estimates of reliability under alternative analytic conditions.
(DOCX)
