# Supplementary material for: Reliability of durometry to assess firmness of calcinosis lesions in Juvenile and adult dermatomyositis
Source: PLoS One. 2026 Mar 23;21(3):e0343708. doi: 10.1371/journal.pone.0343708 (PMC13008098; doi:10.1371/journal.pone.0343708)
Supplement: S1 Fig — Similar make and model of the handheld digital durometer (Rex Gauge 1600 Type DD-4-00) utilized for quantitative assessment of calcinosis firmness. (DOCX) [file pone.0343708.s001.docx]

**Supplemental Figure 1. Image of Handheld Digital Durometer.** Similar make and model of the handheld digital durometer (Rex Gauge 1600 Type DD-4-00) utilized for quantitative assessment of calcinosis firmness.
