# Supplementary material for: Reliability of durometry to assess firmness of calcinosis lesions in Juvenile and adult dermatomyositis
Source: PLoS One. 2026 Mar 23;21(3):e0343708. doi: 10.1371/journal.pone.0343708 (PMC13008098; doi:10.1371/journal.pone.0343708)
Supplement: S1 Form — Categorization of calcinosis lesions by type and anatomic location, were recorded in this Sentinel Lesion Form. (DOCX) [file pone.0343708.s002.docx]

**Supplemental Form 1:**

**Calcinosis Type- Sentinel Lesion Form**

**Subject’s ID number: ____________ ASSESSOR: _______________________**

**Date Assessed: _____________ Assessment number: ______**

Patient’s diagnosis: ___Adult ___Juvenile ___DM ___PM ___ Overlap myositis: specify_________ Other: ________

Please report based on physical exam for all assessments


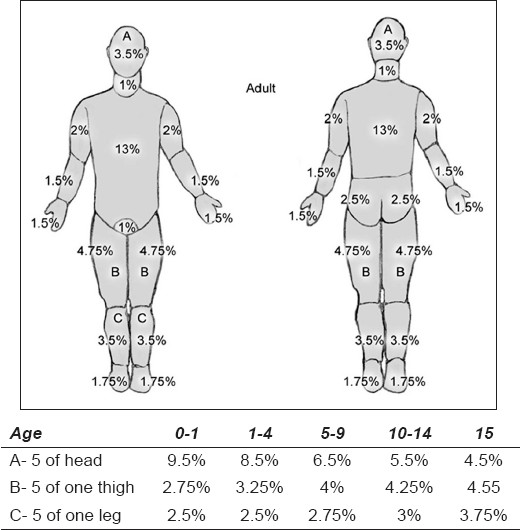
Diagram to mark sentinel lesion locations. Also label with lesion A, anterior or P, Posterior, circle predominant location for each lesion

1. Head (3.5%)*
2. Neck (1%)
3. Torso (13%)
4. Back (13%)
5. Right Upper Arm (2%)
6. Left Upper Arm (2%)
7. Right Elbow (5%)
8. Left Elbow (5%)

**5%**

55

**5%**

55

**5%**

55

**5%**

55

1. Right Forearm (1.5 %)
2. Left Forearm (1.5%)
3. Right Wrist (1.5%)
4. Left Wrist (1.5%)
5. Right Buttocks (2.5%)
6. Left Buttocks (2.5%)
7. Genitalia (1%)
8. Right Thigh (4.75%)*
9. Left Thigh (4.75%)*
10. Right Calf (3.5%)*
11. Left Calf (3.5%)*
12. Right Foot (1.75%)
13. Left Foot (1.75%)

**For a child, use the table below for the head, thigh or distal lower extremity (Anterior or posterior portion)**

| **Region** |  | **5-9 years** | **10-14 years** | **15 years** |
| --- | --- | --- | --- | --- |
| A | ½ of head (anterior or posterior) | 6.5% | 5.5% | 4.5% |
| B | ½ of 1 thigh (anterior or posterior) | 4% | 4.25% | 4.55% |
| C | ½ of lower leg (anterior or posterior) | 2.75% | 3% | 3.75% |

**Glossary**

**Anatomic landmarks:**

The demarcation between anterior and posterior is the midway point between the anterior and posterior of the body.

**Torso:** Laterally it is demarcated by a line drawn from the acromion process anteriorly and posteriorly to the upper body of the axilla and its inferior border is an area defined by the inguinal ligament anteriorly and the gluteal folds posteriorly. This area would include the chest, abdomen, head, neck, back, part of the axilla, and gluteal region.

**Upper Extremity:** Superiorly and medially demarcated by a line drawn from the acromion process anteriorly and posteriorly to the upper body of the axilla. This area would include part of the axilla, the upper arm, forearm, and hand.

**Lower Extremity:** Superiorly bordered by the area defined by the inguinal ligament anteriorly and the gluteal folds posteriorly. It includes the whole of the extremity distal to this demarcation. This area would include the thigh, calf, and foot.

**Axilla:** It is bordered superiorly by the outer border of the first rib, the superior border of the scapula, and the posterior border of the clavicle. The lateral border is the long head of the biceps brachii. The inferior border is the most inferior skin connecting the torso to the upper extremity.

**Elbow:** This region is indicated by a line drawn from the upper edge of the olecranon around to the antecubital fossa. It is considered involved if there is calcinosis crossing this line or within 1 inch of it superiorly or inferiorly.

**Inguinal Ligament:** Area that sits over the inguinal ligament which is a band running from the anterior iliac spine to the pubic tubercle.

**Knee**: Calcinosis is said to be at the knee if it involved the lower extremity at a height between the upper and lower borders of the patella whether the area involved is anterior or posterior.

**Gluteal Region:** Posterior area inferior to the iliac crest, superior to the gluteal fold, and separated into right and left sections by the intergluteal cleft.

**Additional Descriptors:**

**Hard:** Very solid to the touch, rock like.

**Wooden**: Softer to touch than a hard lesion with some give upon palpation, like a piece of wood.

**Fluctuant**: By palpation, feels as if it contains a gel

**Liquefied**: By palpation, feels as if it contains a liquid.

**Tender to palpation**: Elicits pain from the patient when pressure is applied to the area in question.

**Red**: Appears red or purple coloured.

**Warm**: Is hotter to touch compared to the surrounding unaffected skin.

**Swollen**: Abnormally enlarged.

**Ulceration**: Formation of an open lesion in the dermis or deeper.

**Draining:** A substance is seen actively exuding from the skin that can be of any consistency from liquid to chalk like to pure solid.

**Lipoatrophy**: Loss of fat in the affected area.

**Hyperpigmentation**: Darkening of skin color.

**Hypopigmentation**: Loss of skin color.

**Contracture:** Fixed limitation in the normal range of motion of joints due to the shortening or hardening of the involved muscles, ligaments, or tendons.

**Sclerosis**: localized hardening or stiffening of the skin.

**Range of motion:** Normal motion of a joint. Abbreviated ROM.

**Other**: Any other findings felt important about the patient’s calcinosis. For example, this can include such findings as skin sclerosis (localized hardening of the skin).

**Durometry Assessment**

Subject’s ID number: ______________________________

Assessor: ______________________________________

Date of assessment (mm/dd/yy): _____________________

Assessment number: _____________________________

**Assess durometry of up to 5 calcinosis lesions and corresponding areas free of calcinosis,**

**either at the same site as the calcinosis lesions or on the opposite side of the body, but in**

**same location**

**Area 1** ☐Calcinosis ☐Control Picture Number (if picture taken): _____________

Location: _________________________________________________________________________

_________________________________________________________________________________

Measurement #1________ Measurement #2________ Measurement #3________

Matches to Area Number(s):__________________________________________________________

**Area 2** ☐Calcinosis ☐Control Picture Number (if picture taken): _____________

Location: _________________________________________________________________________

_________________________________________________________________________________

Measurement #1________ Measurement #2________ Measurement #3________

Matches to Area Number(s): __________________________________________________________

**Area 3** ☐Calcinosis ☐Control Picture Number (if picture taken): _____________

Location: _________________________________________________________________________

_________________________________________________________________________________

Measurement #1________ Measurement #2________ Measurement #3________

Matches to Area Number(s): __________________________________________________________

**Area 4** ☐Calcinosis ☐Control Picture Number (if picture taken): _____________

Location: _________________________________________________________________________

_________________________________________________________________________________

Measurement #1________ Measurement #2________ Measurement #3________

Matches to Area Number(s): __________________________________________________________

**Area 5** ☐Calcinosis ☐Control Picture Number (if picture taken): _____________

Location: _________________________________________________________________________

_________________________________________________________________________________

Measurement #1________ Measurement #2________ Measurement #3________

Matches to Area Number(s): __________________________________________________________

**Area 6** ☐Calcinosis ☐Control Picture Number (if picture taken): _____________

Location: _________________________________________________________________________

_________________________________________________________________________________

Measurement #1________ Measurement #2________ Measurement #3________

Matches to Area Number(s): __________________________________________________________

**Area 7** ☐Calcinosis ☐Control Picture Number (if picture taken): _____________

Location: _________________________________________________________________________

_________________________________________________________________________________

Measurement #1________ Measurement #2________ Measurement #3________

Matches to Area Number(s): __________________________________________________________

**Area 8** ☐Calcinosis ☐Control Picture Number (if picture taken): _____________

Location: _________________________________________________________________________

_________________________________________________________________________________

Measurement #1________ Measurement #2________ Measurement #3________

Matches to Area Number(s): __________________________________________________________

**Area 9** ☐Calcinosis ☐Control Picture Number (if picture taken): _____________

Location: _________________________________________________________________________

_________________________________________________________________________________

Measurement #1________ Measurement #2________ Measurement #3________

Matches to Area Number(s): __________________________________________________________

**Area 10** ☐Calcinosis ☐Control Picture Number (if picture taken): _____________

Location: _________________________________________________________________________

_________________________________________________________________________________

Measurement #1________ Measurement #2________ Measurement #3________

Matches to Area Number(s): __________________________________________________________
