# Supplementary material for: Reliability of durometry to assess firmness of calcinosis lesions in Juvenile and adult dermatomyositis
Source: PLoS One. 2026 Mar 23;21(3):e0343708. doi: 10.1371/journal.pone.0343708 (PMC13008098; doi:10.1371/journal.pone.0343708)
Supplement: S1 Table — Detailed anatomic site responses from the Sentinel Lesion Form were reviewed and combined into harmonized categories to facilitate downstream analyses. This table summarizes each collapsed site grouping and its component locations. (DOCX) [file pone.0343708.s003.docx]

**Supplementary Table 1. Collapsed anatomic sites derived from Sentinel Lesion Form.** Detailed anatomic site responses from the Sentinel Lesion Form were reviewed and combined into harmonized categories to facilitate downstream analyses. This table summarizes each collapsed site grouping and its component locations.

| **Area Code** | **Category** |
| --- | --- |
| 11, 11A, 11P, 12, 12A, 12P | Upper Neck/Clavicle |
| 13, 13A, 13P 14, 14A, 14P | Back/Torso |
| 15, 15A, 15P, 16, 16A, 16P | Upper Arms Bilaterally |
| 19, 19A, 19P, 20, 20A, 20P | Forearms Bilaterally |
| 17, 17A, 17P, 18, 18A, 18P | Elbows |
| 21, 21A, 21P, 22, 22A, 22P | Hands/Wrists |
| 23, 23A, 23P, 24, 24A, 24P | Buttocks |
| 26, 26A, 26P, 27, 27A, 27P | Thigh |
| 28, 28A, 29, 29A | Anterior Calf |
| 28P, 29P | Posterior Calf |
| 30, 30A, 30P, 31, 31A, 31P | Foot |
