# Supplementary material for: Reliability of durometry to assess firmness of calcinosis lesions in Juvenile and adult dermatomyositis
Source: PLoS One. 2026 Mar 23;21(3):e0343708. doi: 10.1371/journal.pone.0343708 (PMC13008098; doi:10.1371/journal.pone.0343708)
Supplement: S2 Table — This table presents intra-rater intraclass correlation coefficients (ICCs) and corresponding 95% confidence intervals for calcinosis assessments stratified by density characteristics in patients with dermatomyositis and juvenile dermatomyositis (DM/JDM). (DOCX) [file pone.0343708.s004.docx]

**Supplementary Table 2: Intra-rater ICCs and 95% CI for calcinosis assessments by density characteristics for DM/JDM patients.** This table presents intra-rater intraclass correlation coefficients (ICCs) and corresponding 95% confidence intervals for calcinosis assessments stratified by density characteristics in patients with dermatomyositis and juvenile dermatomyositis (DM/JDM).

| **Intra-rater**  **ICC (95% CI)** | **Hardened**  **Only**  **N = 90 / 169** | **Wooden**  **Only**  **N = 12 / 169** | **Fluctuant/Liquified Only**  **N = 7 / 169** | **All Characteristics**  **N = 12 /169** | **Hardened + Woodened**  **N = 40 / 169** | **Wooden + Fluctuant/Liquified**  **N = 8 / 169** |
| --- | --- | --- | --- | --- | --- | --- |
| All Locations | 0.94 (0.92, 0.96) | 0.89 (0.73, 0.96) | 0.79 (0.46, 0.96) | 0.95 (0.87, 0.98) | 0.93 (0.89, 0.96) | 0.92 (0.76, 0.98) |

^*^Of 244 primary rater calcinosis assessments, lesion characteristics were available on 169/244 assessments

Abbreviations: DM (Dermatomyositis); JDM (Juvenile Dermatomyositis); ICC (Intraclass Correlation Coefficient); 95% CI (Confidence Interval)
