# Supplementary material for: Reliability of durometry to assess firmness of calcinosis lesions in Juvenile and adult dermatomyositis
Source: PLoS One. 2026 Mar 23;21(3):e0343708. doi: 10.1371/journal.pone.0343708 (PMC13008098; doi:10.1371/journal.pone.0343708)
Supplement: S3 Table — This table summarizes sensitivity analyses of inter-rater agreement for calcinosis and control assessments across specific anatomic locations in DM/JDM participants, providing estimates of reliability under alternative analytic conditions. (DOCX) [file pone.0343708.s005.docx]

**Supplementary Table 3: Sensitivity analysis for inter-rater reliability of calcinosis and control assessments by Anatomic Location for DM/JDM patients.**

| **Anatomic Location^^,1^** | **Inter-rater Assessments (n=176 pairings)** | **Inter-rater**  **ICC^2^ (95% CI)** | ***Sensitivity***  **Inter-rater**  **ICC^3^ (95% CI)** |
| --- | --- | --- | --- |
| **Calcinosis Location** |  |  |  |
| Upper Neck/Clavicle | 2 | -- | -- |
| Back/Torso | 15 | 0.48 (0.13, 0.76) | 0.37 (0.04, 0.69) |
| Upper Arms | 27 | 0.76 (0.59, 0.87) | 0.65 (0.43, 0.81) |
| Forearms | 9 | 0.64 (0.25, 0.89) | 0.66 (0.27, 0.90) |
| Elbows | 9 | 0.85 (0.61, 0.96) | 0.75 (0.43, 0.93) |
| Hands/Wrists | 4 | -- | -- |
| Buttocks | 3 | -- | -- |
| Thigh | 26 | 0.32 (0.05, 0.58) | 0.29 (0.03, 0.56) |
| Anterior Calf | 10 | 0.30 (-0.16, 0.73) | 0.18 (-0.26, 0.66) |
| Posterior Calf | 6 | 0.79 (0.40, 0.96) | 0.67 (0.19, 0.94) |
| Foot | 2 | -- | -- |
|  |  |  |  |
| **Control Location** |  |  |  |
| Upper Neck/Clavicle | 1 | -- | -- |
| Back/Torso | 9 | 0.65 (0.22, 0.90) | 0.59 (0.14, 0.88) |
| Upper Arms | 16 | 0.47 (0.13, 0.75) | 0.51 (0.18, 0.77) |
| Forearms | 9 | 0.83 (0.55, 0.95) | 0.70 (0.31, 0.91) |
| Elbows | 4 | -- | -- |
| Hands/Wrists | 0 | -- | -- |
| Buttocks | 2 | -- | -- |
| Thigh | 12 | 0.30 (-0.12, 0.70) | 0.34 (-0.08, 0.72) |
| Anterior Calf | 5 | 0.56 (-0.03, 0.93) | 0.35 (-0.28, 0.89) |
| Posterior Calf | 4 | -- | -- |
| Foot | 1 | -- | -- |

^^^57 patients and 5 raters provide data for 129 patient-provider interactions, on 11 unique locations and totaling 709 durometry measurement assessments, both calcinosis and control

^1^Intra-rater and inter-rater ICCs calculated when number of assessments ≥ 5

^2^Repeated intra-rater durometry measures were averaged and these averages were compared between corresponding main and secondary raters, when available, for inter-rater ICC evaluation

^3^First intra-rater durometry measures were compared between corresponding main and secondary raters, when available, for inter-rater ICC evaluation

^*^245 assessments had no corresponding primary *and* secondary rater(s); 176 primary assessments had 1+ corresponding secondary assessments for inter-rater ICC calculations (n=176 inter-rater pairings, comprised of 464 primary and secondary assessments)

Abbreviations: DM (Dermatomyositis); JDM (Juvenile Dermatomyositis); ICC (Intraclass Correlation Coefficient); 95% CI (Confidence Interval)
